# Supplementary figures and images for: Data-driven evaluation of the Boston marathon qualifying times
Source: PLoS One. 2023 Apr 19;18(4):e0283851. doi: 10.1371/journal.pone.0283851 (PMC10115302; doi:10.1371/journal.pone.0283851)

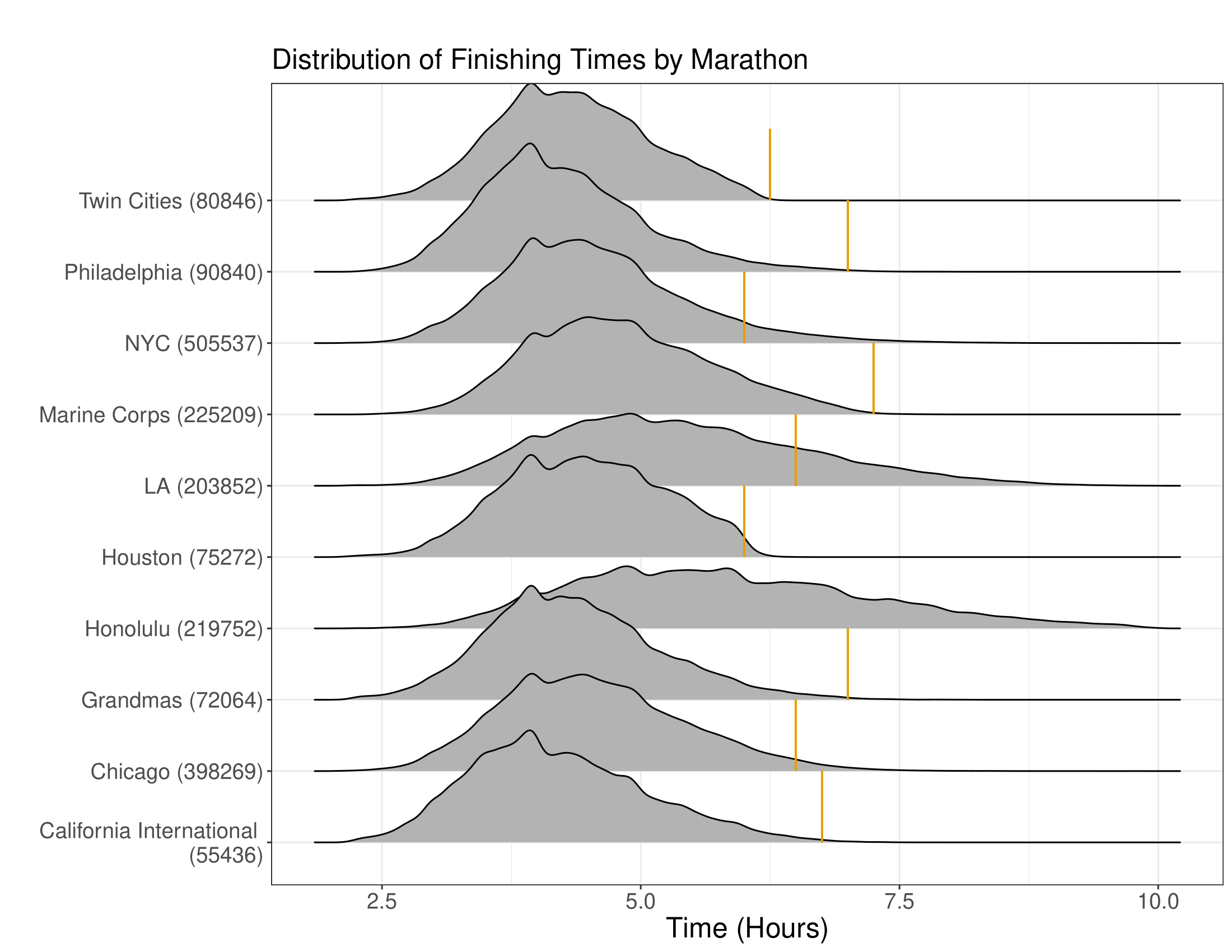

Supplement: S1 Fig — The total number of runners observed in each marathon is shown in parentheses. The orange vertical line indicates the cut-off time for each marathon. Some marathons are strict with their cut-off times while others are more lenient. An interesting feature to note, in each marathon except for Honolulu we see a bump right around the 4 hour mark, a popular goal time for many runners. (TIF) [file pone.0283851.s001.tif]

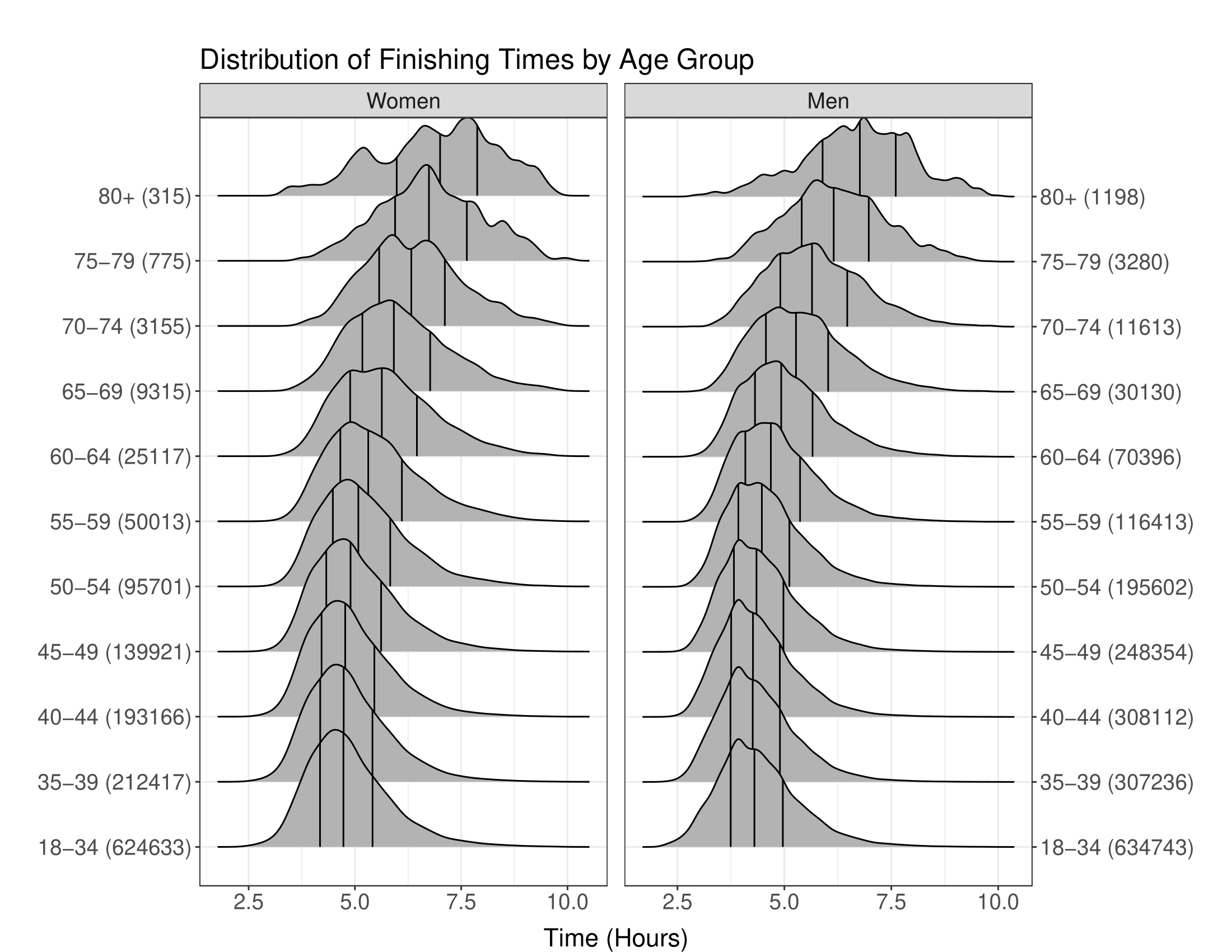

Supplement: S2 Fig — The number of runners in each age group and gender are shown in parentheses. The distribution of times does not change much between the 18–34, 35–39, and 40–44 age groups. We then see a shift towards slower times in each subsequent age group, particularly in the oldest age groups. (TIF) [file pone.0283851.s002.tif]

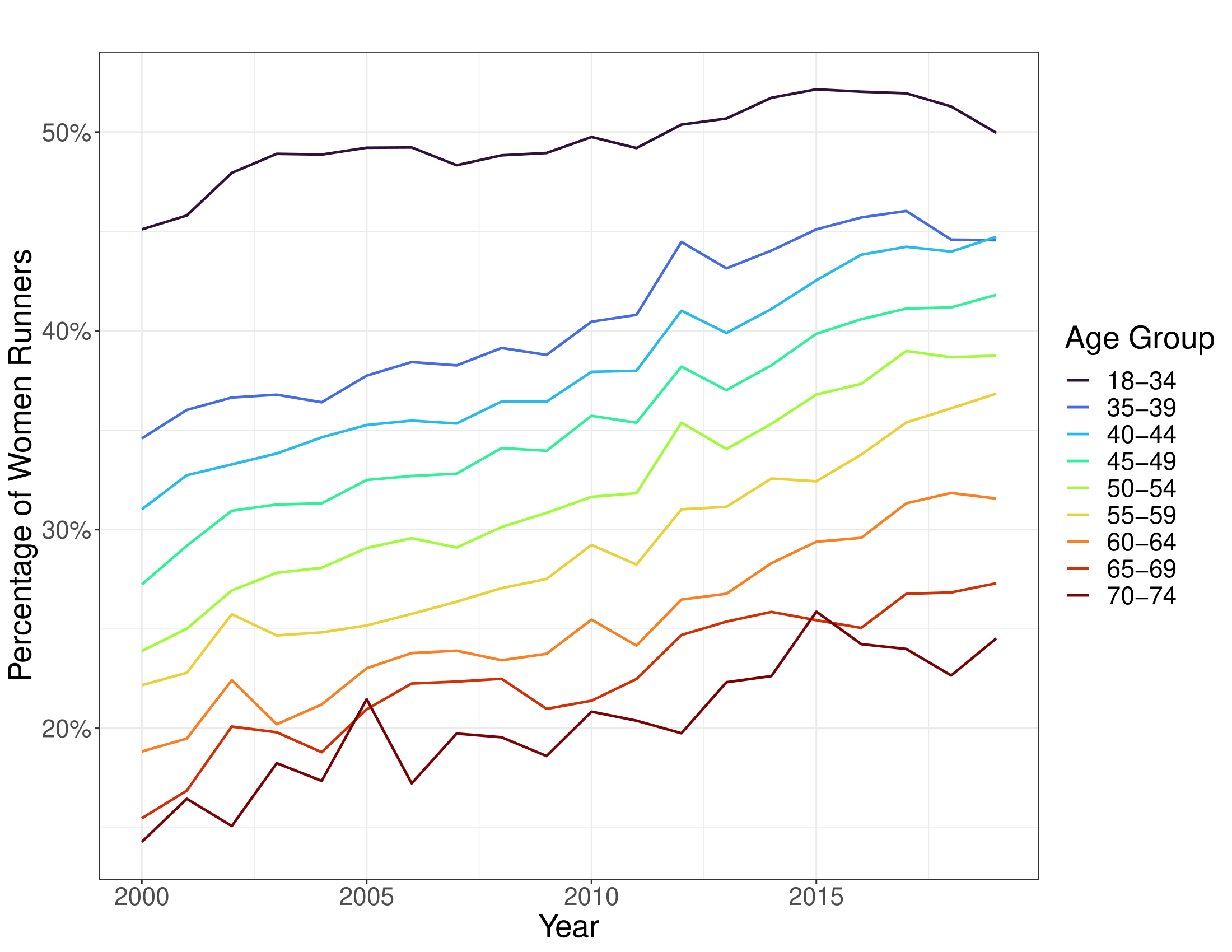

Supplement: S3 Fig — The 18–34 age group increased towards 50% and then appears to have leveled off around that 50% split. All other age groups are increasing on average each year. We observe a sharper increase in the percentage of Women in many age groups in 2012 due to New York not being held that year. New York has a lower percentage of women runners than most other marathons so it’s exclusion leads to the spike observed. (TIF) [file pone.0283851.s003.tif]
