# Supplementary material for: Data-driven evaluation of the Boston marathon qualifying times
Source: PLoS One. 2023 Apr 19;18(4):e0283851. doi: 10.1371/journal.pone.0283851 (PMC10115302; doi:10.1371/journal.pone.0283851)
Supplement: S1 Table — (PDF) [file pone.0283851.s004.pdf]

S1 Table Number of participants by marathon and age group.

Marathon	18-34	35-39	40-44	45-49	50-54	55-59	60-64	65-69	70-74
California International	29446	16473	17034	14615	10039	5834	2826	1142	445
Chicago	309526	120824	108532	77875	51767	26812	11834	3998	1136
Grandmas	53230	18670	16318	12825	9742	5595	2916	1148	351
Honolulu	160459	49998	45078	40172	39417	30656	27820	14112	5799
Houston	37841	20885	20477	16735	11933	6724	3150	1118	323
LA	137380	49446	46316	38503	28140	17511	9571	4544	1921
Marine Corps	148791	65054	61210	47035	33176	18473	8890	3403	1067
NYC	252159	131649	144089	108642	85555	42938	23259	8151	3153
Philadelphia	68995	24311	22413	17051	11180	6117	2572	846	237
Twin_Cities	61549	22343	19811	14822	10354	5766	2675	983	336
