# Supplementary material for: Data-driven evaluation of the Boston marathon qualifying times
Source: PLoS One. 2023 Apr 19;18(4):e0283851. doi: 10.1371/journal.pone.0283851 (PMC10115302; doi:10.1371/journal.pone.0283851)
Supplement: S2 Table — Differences in cut-off times may be responsible for the variation in the proportion of qualifiers across different marathons. Also, each marathon has different levels of enforcement with cut-off times which could impact the distribution of finishers. (PDF) [file pone.0283851.s005.pdf]

S2 Table. Cut-Off Times and Average Number of Participants by Marathon. Differences in cut-off times may be responsible for the variation in the proportion of qualifiers across different marathons. Also, each marathon has different levels of enforcement with cut-off times which could impact the distribution of finishers.

Marathon	Cut-off Time	Average # of Participants
California International	6:45	5059
Chicago	6:30	36153
Grandmas	7:00	6428
Honolulu	None	21307
Houston	6:00	6081
LA	6:30	19046
Marine Corps	7:15	19565
New York	6:00	42853
Philadelphia	7:00	7938
Twin Cities	6:15	7854
