# Supplementary material for: Data-driven evaluation of the Boston marathon qualifying times
Source: PLoS One. 2023 Apr 19;18(4):e0283851. doi: 10.1371/journal.pone.0283851 (PMC10115302; doi:10.1371/journal.pone.0283851)
Supplement: Attachment — Submitted filename: Response to Reviewers.pdf [file pone.0283851.s006.pdf]

Reply to the Reviewers

Re: Manuscript ID

"Response to Reviewer comments for: Investigating the Fairness of the Boston Marathon qualifying times"

Laura Albrecht[1], *Ross Ring-Jarvi*[1], *Dorit Hammerling*[1]

PLOS One

1 Department of Applied Mathematics and Statistics, Colorado School of Mines, Golden, CO, United States of America

We thank the reviewers and editor for their critical assessment of our work. We address their comments point by point.

Reviewer #1, comment #1

I believe reviewer 2 makes a legitimate case for the use of fairness. While I believe you have defined the word, I question whether fairness is relevant in a scientific paper. I believe you should reposition your paper as evidence-based for qualifying times. This is a more direct way to direct your argument; Are the current standards evidence-based? It doesn't appear so. Is your approach better? I can't say, but we should establish standards based on the evidence, not arbitrary fixed numbers. That is the case the authors should make and a suggestion on how to get there!

Our response #1.1

We think this is an excellent suggestion. We agree presenting our method as an evidence-based alternative is clearer and less subjective. We have repositioned our paper as the editor suggested and have removed the discussion of the word fairness.

Reviewer #1, comment #2

There is inconsistent grammar, language use (like many in areas where present not past tense is used). The paper feels disjointed as if written by different individuals and then combined without regard to flow.

Our response #1.2

We have edited the paper to make sure the tense is consistent throughout each section and have added some transitions to try and make the paper flow better.

Reviewer #1, comment #3

There are insufficient references for a paper of this magnitude. In particular, much of the (limited) discussion is more extended conclusions and speculation and lacks any meaningful literature to support it. I recommend several references to review: Bagley DOI: 10.1111/sms.13404 Tanaka DOI: 10.1113/jphysiol.2007.141879 Nicolaidis doi: 10.3390/ijerph16101777 Waldvogel doi: 10.3390/ijerph16132377 https://runrepeat.com/state-of-running https://runnerclick.com/marathon-finishing-times-study-and-statistics/

Our response #1.3

Thank you so much to the editor for taking the time to include these references as a starting point. We have expanded the discussion to incorporate more references, including several of those suggestions, that are relevant to our hypothesis and results.

Reviewer #1, comment #4

The introduction is extensive and well written but should end with a clear purpose and hypotheses.

Our response #1.4

Thank you. An additional paragraph has been added to the end of the introduction to make our purpose more clear and help ease the transition into the methods section.

Reviewer #1, comment #5

The Methods are very confusing and poorly structured, and should be written in the past tense. The general writing is completely different from the intro and much of the methods are actually results. I recommend streamlining and reorganizing to clearly identify who the participants were, where the data came from, how it was analyzed with details on stats, and specific criteria for exclusion of data. I envision much of the data with figures should move to results.

Our response #1.5

Thank you for bringing this to our attention. We have revised the methods section to try and make it more clear and hope the tone fits better with the rest of the paper now. Most of the figures and exploratory data analysis have been moved to either the results section or the supplemental information.

Reviewer #1, comment #6

Results are extensive but should be streamlined and flow based on the story you with to tell in the discussion.

Our response #1.6

We have updated the results section to to better focus our story. Fig 6 has been moved to the supplemental information section.

Reviewer #1, comment #7

Figures are at times confusing with superfluous text on the figures. Titles need to be concise where possible and clearly identify what the figure is showing without excess story or text. I'm just having a hard time following the story you're telling. I recommend identifying the key 3 or 4 figures and moving the rest to supplements to tell your story.

Our response #1.7

We have updated all the figure captions to be more concise. We really appreciate the suggestion to identify the key figures. This really helped us focus our story and improved the flow of our paper. We have moved four of the figures into the supplemental section. While we believe these figures give interesting insight into our data set, we agree the supplemental section is probably a better place to include them.

Reviewer #1, comment #8

The discussion is inadequate for the paper and data you present. Two big paragraphs do little to help the paper and largely just try to tell us how much better your optimized times are. However, you haven't really made that case, and really should leave it to the conclusion and future rec's for research. It also needs to lean into supporting references. I suggest: Restating the purpose and hypotheses then reviewing what you found. Then each subsequent paragraph targets a single finding. An additional paragraph can cover unexpected findings and then perhaps one more paragraph to note the limits of this study and future recommendations.

Our response #1.8

Thank you for this very specific outline on how to improve our discussion. We have thoroughly reorganized the discussion to follow this outline. We have expanded our discussion to include more references and added a discussion about the limitation of our work and potential directions of future work.

Reviewer #1, comment #9

Now hit the conclusion with the overarching findings and message. Avoid over speaking your results.

Our response #1.9

We have revised the conclusion in this way. We truly thank you for taking the time to provide such detailed and useful suggestions for each section of our paper.

Reviewer #2, comment #1

I am curious on why those ten races were selected for data analysis. It is not noted in the methods. When looking at the top qualify races for Boston, several of them are not even listed (e.g. Honolulu, Houston, LA). Top Qualifying Races — Boston Athletic Association (baa.org). Please provide more information on this.

Our response #2.1

Thank you for bringing it to our attention that we failed to explain our choice of marathons. We chose these 10 marathons to get a good geographic sample of marathons that are 10 of the top 15 largest marathons in the US. Additionally, all marathons except for Honolulu are listed as top qualifying races for Boston. We have added an explanation of this in the Data section.

Reviewer #2, comment #2

This reviewer suggests providing a shorter summary after each Figure. The current summaries are very lengthy and are what is exactly in the text of the article. In addition, the color coding is helpful but many readers will be looking at this article in black/white (especially if print). This reviewer would suggest looking at different ways to express the data so can be viewed by all.

Our response #2.2

Thank you for the suggestion. The figures summaries have been shortened on all figures. The color choices in our figures all come from color blind friendly color palettes and as this is an online only journal, we believe they should be readable to readers.

Reviewer #2, comment #3

Since, this study looked at data prior to the pandemic, do the authors feel that this has now changed the outcome of their data. Boston has now opened up the race for more athletes to be able to run. This might be something to mention in the discussion.

Our response #2.3

This is an interesting point we hadn't considered yet. We have added a paragraph in the discussion regarding the effect of the pandemic on marathons and how this will impact future data. We only collected data on and explored marathons prior to the pandemic but hypothesize it may take a few years for road racing to rebound to pre-pandemic popularity. For the first time since 2013, all runners who qualified and entered Boston were accepted to run it this year. The field size for the 2022 race was the same as the previous 5 years before the pandemic. We assume the abundance of cancelled or postponed races in 2020 and 2021, which limited the opportunities to achieve a qualifying time, may have been a big contributing factor to the easier entrance criteria this year. The effect of the pandemic on road race participation across genders and age groups will be interesting to look at in the coming years.

Reviewer #3, comment #1

First, I believe that if authors want to evaluate fairness, they should include a proper definition of the term and how this affected their research question. Fairness in sports is a rather complicated matter, involving several other factors that were not considered in the study and not discussed properly. In my opinion, the study simply aimed to analyse whether the current Boston qualifying times include a similar percentage of women and men, and whether the times could be changed by applying a mathematical model in a way that the difference in proportion of qualifiers across genders is smaller (although the authors do not report how much this difference is).

Our response #3.1

We agree that the discussion of fairness is complicated and have instead reframed our paper, as suggested by the editor, away from fairness. In Fig 4 we show a side by side bar plot of the results of the proportion test at the current Boston qualifying times and our optimized times to try and show that our optimized times lead to a more equal proportion qualifiers. We have edited the explanation of this plot to try and highlight that result more explicitly. We have also added a row to Table 2 showing the difference in proportions between men and women across all races at the current and optimized times.

Reviewer #3, comment #2

Second, it is not clear from the introduction and the aims of the study what is the current differences in the number of women and men that qualify in each race. This is a piece of very important information, that would allow us to know if there is indeed a large difference in the proportion of qualifiers across genders.

Our response #3.2

Thank you and we agree we skimmed over detail and it needs to be more clear. In the second row of Fig 1 we show the proportion of men and women that qualify in our data in each age group. Other than the 18-34 age group which has a slightly higher percentage of men qualifying than women, women qualify at a higher rate for all ages 50 and under. For age groups 55 and over, the percentage of men that qualify is greater and the gap between the proportion of men and women qualifying increases in each subsequent age group. This explanation has been added to the Data section.

Reviewer #3, comment #3

Also, the methods section is quite complicated to follow and there is some information that is not relevant to the aims of the study. For example, the authors surprisingly collected data from 10 different other marathons, and it is not clear why they did that. I believe most of the data that were analysed and presented are not relevant to the aims of the study which was to analyse whether the Boston qualifying times include a similar proportion of runners across genders. I believe the authors should then focus on the "Proportion Analysis" assessing the Boston qualifying times and how changing it would minimize the difference in the proportion of qualifiers across each race. The authors also included in the methods sections, several results from their analysis that should be included only in the "Results" section.

Our response #3.3

Thank you for this feedback. We have edited the methods section to hopefully make it easier to follow. We have also moved any results in the methods section into the results section. As described above, we have added an explanation to why these 10 marathons were chosen. Many of the exploratory data pieces were moved to the supplemental information as we think it may be of interest to reader, especially those in the running community, but agree it detracts from the story we are trying to tell.

Reviewer #3, comment #4

Finally, the "Discussion" section does not represent all the data that was analysed. The authors previously presented the results from several different analyses, yet the discussion had only two paragraphs and only included one reference. The authors should aim to discuss the results of their study comparing it with previous published studies, making sure that all the data that was analysed, is discussed as well.

Our response #3.4

Thank you for this suggestion. We have expanded the discussion section to ensure all of the results

presented are included. The discussion has been expanded as described in response to the editor's comments to include a more thorough comparison with previous literature.
