# Supplementary material for: Data-driven evaluation of the Boston marathon qualifying times
Source: PLoS One. 2023 Apr 19;18(4):e0283851. doi: 10.1371/journal.pone.0283851 (PMC10115302; doi:10.1371/journal.pone.0283851)
Supplement: Attachment — Submitted filename: BQ_PLOS_Response_Letter_2.pdf [file pone.0283851.s008.pdf]

Reply to the Reviewers

Re: Manuscript ID

"Response to Reviewer comments for: Investigating the Fairness of the Boston Marathon qualifying times"

Laura Albrecht[1], Ross Ring-Jarvi[1], Dorit Hammerling[1]

PLOS One

1 Department of Applied Mathematics and Statistics, Colorado School of Mines, Golden, CO, United States of America

We thank the reviewers and editor for their critical assessment of our work. We address their comments point by point.

Reviewer #1, comment #1

A major issue is the sample and sampling method. The gold standard is random sampling, yet the authors say they chose by 'geographic spread' – is this good (probably not)? Is it random (definitely not)? Usually, in sampling, if trying to make inference about a population (such as the USA), then geographically representative proportions could be targeted, e.g., proportions of population by State. This would involve heavier weighting of more populous states. One might only want a uniformly geographic sample, if say, we were sampling 50 senators on how they would vote on something. If random is not possible would some other matching or appropriate sampling method be better? These seem to be the 'most common races' to qualify for Boston, but how common? Can we know what % of qualifiers this would represent? What is the population of potential qualifiers? Are they actually mostly in States like California, Illinois, NY, etc? This is just not clear. They then further carry out sensitivity checks by excluding one marathon and seeing the difference to the results. Line 128-34 gives that this might make 1 minute difference – is this significant? Moreover, however, this robustness check merely tests their sample to sensitivity WITHIN THEIR ALREADY (BIASed???) SELECTED SAMPLE. What we'd really like to know is how different this sample is to a population, not relative to itself minus one. They either need to have some way of finding covariates for marathons and marathoners included and excluded and seeing if these are matching or finding more data on dissimilar marathons, or give evidence the marathons excluded are so small they will not matter.

The remedy suggested here is several-fold. Do we know something about runners and where they are from, nationally and in the races selected? Can we know what % of qualifiers come from these races? If this information is available, use it and discuss why this means the sample chosen is good. For example, we might be close to the population here—if more than 2/3s or $\frac{3}{4}$ of qualifiers are already coming from these races, then we're a long way to actually having the population, rather than a sample. If this isn't available, some means of choosing some random races and checking statistically a) how differently they are from the current sample and b) how much the sample might change if they were included.

Our response #1.1

Thank you for the detailed recommendations on how to justify our chosen sample more thoroughly. We know that a random sampling approach would be ideal but due to the difficulty of collecting these race results across multiple years, it just isn't something that we found feasible to do. We instead strategically chose races that both produce a high number of qualifiers each year and had accessible data. The Boston Athletic Association provided us with their internal data for 2018 and 2019 that includes which race each participant used as their qualifying race. The marathons that we selected account for 28.6% of all qualifiers in those years when excluding the Boston Marathon itself. The marathon that produced the highest percent of qualifiers each year (roughly 10%) was the previous year's Boston Marathon, respectively. We chose not to include the Boston Marathon in our analysis since it is an already biased sample of runners that have previously qualified somewhere else in the past. We consider a sample containing over 28% of all qualifiers a reasonable sample but do not claim that it is necessarily fully representative of the entire population. We have incorporated this into our explanation of the data set and have noted that future work could include collecting more race results and repeating this analysis.

The home location of runners in each of the marathons in our data is a little messy but we have done our best at extracting this information. We focus only on runners that live in the US as the formatting for cities/countries for international runners is not consistent across races. Maps of the difference in the distribution of runners in the Boston marathon and our data set are shown in Figure 1. The highest proportion of entrants in the Boston Marathon are from Massachusetts while the highest proportion of entrants in our data are from California. We observe slightly more runner from Minnesota, Illinois, Texas, and Virginia in our data set (all locations in which one or more of the races in our data set took place). Otherwise, the distribution of runners coming from all other states across the US is consistent in our data compared to the Boston marathon data. We have added this to our explanation of the data set.

Reviewer #1, comment #2

There is a pandora's box of issues with regards to 'fairness' and I think still more effort is needed here. The use of the word 'optimal' while in a mathematical sense is not linked to fairness still actually IS linked to some notion of fairness, as the authors have arbitrarily chosen an objective function to minimize – why not

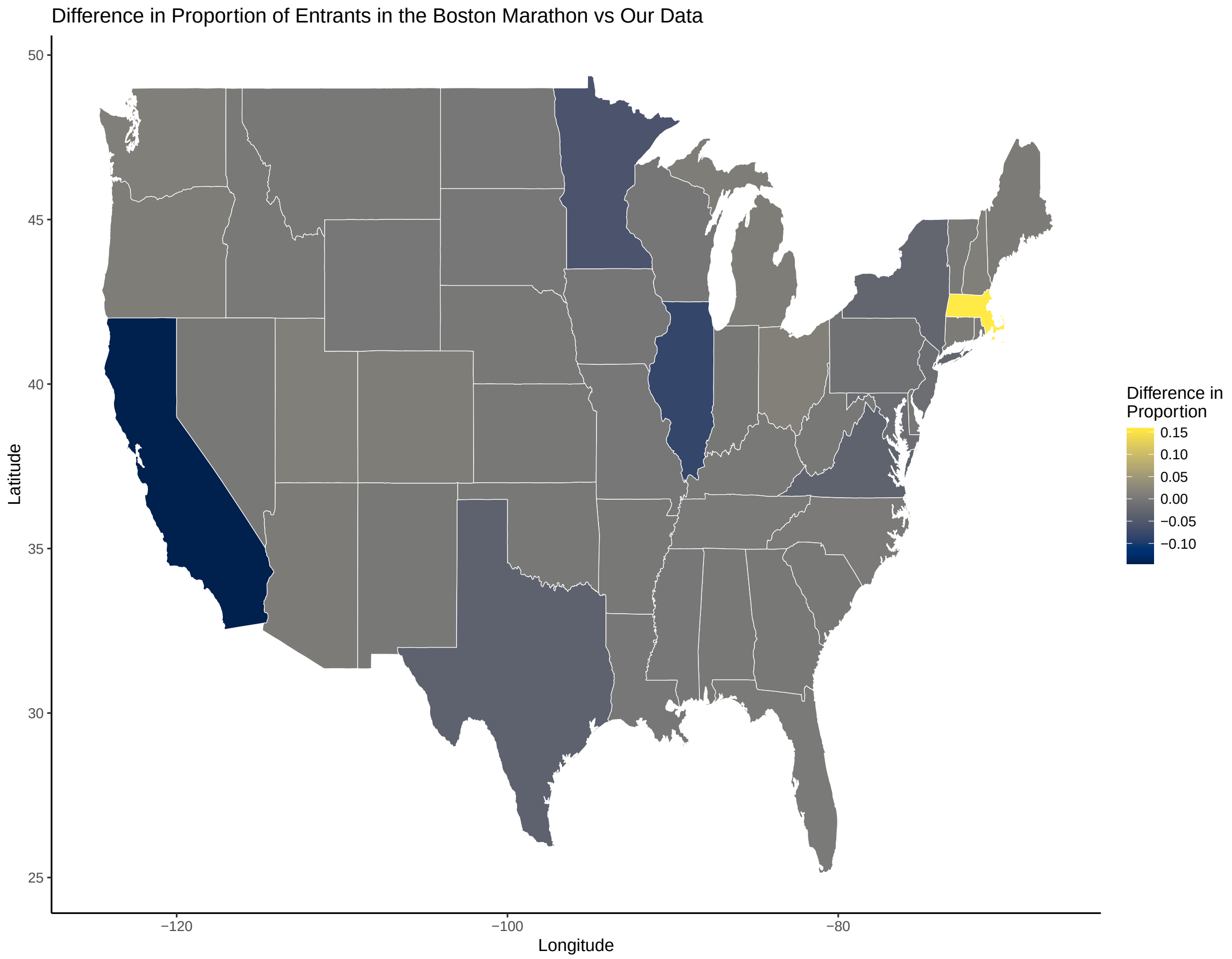

Figure 1: Difference in the proportion of entrants that live in different states in the US between the Boston Marathon and our data set from 2010-2019.

the sum of squared proportional differences? Why not weight by income for age group? Optimized in the pure mathematical sense means a chosen objective function. On the other hand, it can be interpreted more widely to mean 'best' – but who's best? Semantically there is no difference between this looser understanding of 'optimal' and 'fair' or 'best'. They should avoid the use of 'optimized' – and instead use something such as proportional-difference-minimized (or perhaps they can come up with some other way to name their outcome). This is not intractable, nor should the paper get too much into this, but recognition of this and some discussion is needed and to be clear that this is 'optimal' only in the sense of having minimised an arbitrary objective function.

Our response #1.2

We have removed the word 'optimal' from our manuscript. We have updated the use of the word optimize to show we are trying to minimize our objective function which is to make the proportion of men and women similar. We have added a discussion of why we chose this specific objective function and discuss how many potential objective functions could be utilized and note our method could be applied to other objective functions that fit the race organizer's goals and desired distribution of qualifiers.

Reviewer #1, comment #3

To show how this could go in many ways, they seem to indicate their proposed qualifying standard is 'better' if Boston used – why? What is the Boston Marathon's objective function? What should it be? Why not also minimize the proportional differences 'across age groups' for men and women combined, or separately? (In other words, what % of men and women 75-79 qualify relative to say 30-34?) My understanding is that the proposed approach minimises the proportional difference in qualifiers relative to men of the SAME age group—but what if both men AND women find it harder to qualify as they get older? Further, they seem to indicate Boston might not have made their QTs based on evidence—do we know this? –maybe they had evidence that simplicity was better in some cases than (an undefined) fairness? We just don't know.

Our response #1.3

This brings up a great point we had not sufficiently addressed regarding the history of the BAA setting qualifying times. Unlike the organizers of the NYC Marathon who have been more transparent, the BAA has released very little information on their rational behind how they determine their qualifying times for different age groups and genders. When women were first allowed to enter Boston 1972, they had the same qualifying times as men. In 1976 the women's time was changed to be 5 minutes slower than the men's time. In 1987 the fixed 30-minute time difference between men and women was introduced. Despite numerous alterations to the qualifying times based on age since then, the 30-minute time difference has remained the time gap between men's and women's times in all age groups ever since. The 70-74, 75-79, and 80+ age groups were added in 2002, again adopting the same fixed 30-minute time difference that existed at the younger age groups. To the best of our knowledge, the use of this fixed time difference in every age group is not a data-driven decision. We have incorporated this history of Boston qualifying times into our introduction.

Figure 1 shows that the proportion of qualifiers at the older age groups is higher than it is at the younger age groups. Therefore it would seem it is "easier" to qualify at older age groups than younger age groups. Therefore, if we try to find a consistent proportion of qualifiers across all age groups, this will decrease the number of qualifiers in the older age groups and increase the number of qualifiers in the younger age groups. In 2002 the BAA altered the qualifying times to make the times easier for all runners 45+. At this time they also created the 75-79 and 80+ age groups. According to the BAA's website the reason for this change to the 45+ times was to accommodate more participants. It appears that they wanted to specifically accommodate more participants in the older age groups. We assume, based on the BAA's decision in 2003 to make the qualifying times slower in the older age groups, that the BAA wants to encourage participation in the older age groups. Therefore, we chose to keep the proportion of male qualifiers constant. We have added this explanation to our discussion.

Our response #1.3

Reviewer #1, comment #4

The results and proposed changes don't really seem to change that much relative to what the authors claim as their original purpose is. Moreover, they seem to gloss this over, or else they've missed it.

The 'optimal' new times would appear to trade-off 3,792 fewer women qualifying overall, 5,519 lose out on qualifying across the negative categories, but mostly in the 35-39 and 40-44 age cates, all to get 148 more women into 65-74 and 1579 into 18-34? It would appear the main redistribution is towards young women, no? Why is this 'better'? Is it possible I have read this table wrong? In spite of the authors saying that the main thing 'wrong' with the current system being that it would appear women in older age categories are disadvantaged relative to men, their results overall on the face of it don't really seem to bear this out; the main increase is in 18-34 year old women. Now granted in % terms the largest is 70-74 year olds. (Moreover, I don't understand the fact that all the % in the bottom row are negative but the (+/-) signs in the bottom ()'s seem consistent with row 2?. I would have thought a more sophisticated constrained optimisation, holding the total number of women constant (for example), would have been possible.

Our response #1.4

We appreciate this feedback on our results and believe the reviewer raises several good points. Our intention wasn't to necessarily find qualifying times that are different than the current times but just to compare the impact of setting qualifying times with our data-driven approach versus the fixed 30-minute gap. We agree that the proposed changes are fairly small and the current BQ times are overall consistent with the data-driven approach. We have updated the manuscript to better state this in our discussion.

There are many ways you could choose to optimize the qualifiers here. Our aim was to investigate the proportion of women that qualify relative to men in each age group. Therefore, we did not want to constrain the number of women qualifying as this would no longer allow for a direct comparison in each age group. In Fig 1 of the manuscript, we see the proportion of women qualifying for Boston is higher than for men at the 35-49 age groups and at the 60+ age groups the proportion of men qualifying is much higher than women. Since there are more women in the 35-49 age groups than 60+ it makes sense that the total number of women qualifying would decrease. While it is possible to do a constrained optimization holding the number of women more or less constant will favor the current qualifying times since small changes in the qualifying times in the younger age groups causes such a radical difference in the number of qualifiers. Doing such does not allow these difference in proportion between genders to be fully explored. At the current QTs, the proportion of qualifiers at the older age groups is higher than it is at the younger age groups for both men and women (Fig 1). Therefore, we chose to keep the men's times constant because finding a consistent proportion of qualifiers across all age groups would decrease the number of qualifiers in the older age groups and increase the number of qualifiers in the younger age groups. In 2002 the BAA altered the qualifying times to make the times easier for all runners 45 and older to accommodate more participants. Based on this decision, we assume the BAA wanted to specifically increase participation in the older age groups. While the absolute number of qualifiers shifts in favor of the 18-34 age group in our method, the biggest percentage difference, however, comes in the oldest two age groups.

The last row in Table 2 gives the percent difference between the proportion of men and the proportion of women that qualify at the respective qualifying times. A negative value indicates a higher proportion of men qualify relative to women. That is, at the current BQ times the proportion of women that qualify is less than the proportion of men for the 18-34 age group by 3.47%, at the optimized QT the difference is only 0.09%.

Reviewer #1, comment #5

This further also raises the question of whether the overall proportion of women qualifying is 'harder' or easier than for men; some mention and discussion of this should be done. (In other words, do 10% of top women qualify at the top races and 10% of men, roughly?, or is it 10,000 men and 10,000 women, and maybe that's 5% and 10% of some pool of potential qualifiers?). An interesting comparison here would be how the overall number of men and women changed in Boston when Boston changed the qualifying standards. How

do these numbers compare to the proposed new changes? Are there other marathons that have changed similarly.

Our response #1.5

Figure 1 in our paper contains most of this information but we realize the discussion around this figure may not have been robust enough. We appreciate you bringing this to our attention. Also, we have now provided a longer discussion about the history of Boston qualifying times to motivate and explain our choice of objective function which should be helpful to address this point.

We expanded the explanation and discussion of Figure 1 which shows the percentage of men and women qualifying in our data set. In Figure 1 we see that the proportion of men that qualify is slightly higher than women in the 18-34 age group in our data. In the 35-39, 40-44, and 45-49 age groups more women qualify proportionally than men. The proportion of qualifiers is pretty equal between men and women at the 50-54 and 55-59 age groups. However we see at the 60-64, 65-69, and 70-74 age group the proportion of men qualifying is higher and the gap between the proportion or men and women grows in each age group. This was our motivation to find qualifying times that lead to a more equal proportion of qualifiers. This is a complicated problem because as explained previously, while the highest number of runners (and also highest number qualifiers) are in the youngest two age groups, these age group also have the lowest percentage of qualifiers in both genders. So, if we try to find a consistent proportion of qualifiers across all age groups, this will decrease the number of qualifiers in the older age groups and increase the number of qualifiers in the younger age groups. This led us to keep the proportion of men qualifying set and only minimize with regards to the women's times.

We agree a comparison of the impact on the field due to these new time standards would be interesting. Unfortunately, the Boston Marathon has not had a "normal" year since the introduction of the latest time standards due to the pandemic so this isn't feasible yet and something to address in future work.

Reviewer #1, comment #6

The authors then seem to gloss over this fact of the potential large increase in 18-34 women – for example: 1) they don't mention this in the conclusion. 2) At line 187 they state, "Our results are consistent with those in [20] that found the 2013 Boston qualifying standards were too lenient for women 18-54 and too strict for women 55-80." – this seems in contradiction to their results – their results find QTs too strict for 18-34 AND 55-75 women. 3) The conclusions seem to indicate that the +1 minute for the 18-34 group found from the procedure should not be done; but the other proposed changes should be done.

Our response #1.6

We have updated the discussion regarding the 18-34 age group. This one minute difference does cause a large increase in the number of qualifiers but proportionally the change is relatively small. We have made this more clear and have restated our conclusions to include this one minute change in this age group as well.

Reviewer #1, comment #7

At line 166 they seem to be over-interpreting their results: "This is thought to be a result of the disproportionate obligations women still face in child rearing which could result in runners in these age groups being disproportionately comprised [in their training relative to?] of more serious and faster runners [17]." Is this really true? There is just one (seemingly a very weak reference?) The statement would be much better written as "one interpretation suggested this to be a result of the disproportionate. . . .."

Moreover, it seems strange that this claimed child-bearing rationale is given for 35-39, and 40-44 women's getting faster (and skewed distribution). A quick google of this for fertility by mother's age seems to indicate differently—the highest birth rates are for 20-30 years olds.

This seems to be a very charged and unsubstantiated claim which is at odds with basic fertility rate data – it may be true – but the authors need to either address this substantially or take this out.

Our response #1.7

We agree, this was a little speculative and not well supported. We have removed this reference as to the

reason for this decline and have instead focused on the fact the ratio of women/men in our data set decreases drastically at 35+.

Reviewer #1, comment #8

Finally, the conclusions use language that is a bit unclear. "By optimizing the proportion of qualifiers across all marathons and years" Besides the already mentioned suggestion that 'optimal' is little better than 'fair' – what does 'optimising the proportion' mean? What actually was done was minimize proportional differences between gender for each age cohort, no? Some more precise language and description is required here. "Our method allows the qualifying times to be supported by data instead of being set on a sliding scale"; again, do we know the sliding scale wasn't set using data? Say something more precise about what was done – e.g., using the large data set covering x% of all qualifiers from past 10 years (or whatever that should say). "leading to a proportionally more representative group of qualifiers across gender and age." Again, it isn't clear what this means and is seemingly imprecise—representative on what?—we need to have some prior of the 'true' population proportion. Usually, when we say "representative" we mean that a sample is constructed to be proportionally matched on some criteria, such as proportion of males and females, age cohort, in a population—the population proportion is known (e.g., Census data). But this isn't what is going on here – either make the case of why this is 'representative' of a population proportion or use some other language.

Our response #1.8

Thank you for these detailed suggestions. We have removed the optimal/fair wording to be more precise in what we are trying to minimize. The previous feedback has also been incorporated into the conclusion regarding our sample and the current BQ times.

Reviewer #2, comment #1

There is a lot of information that is not relevant to the aims of the study. For example, I believe most of the figures included in the supplemental material are irrelevant and should be excluded from the study.

Our response #2.1

Thank you for this feedback. We have removed one of the figures from the supplemental material. We feel the remaining figures in the supplemental material are helpful to understand the distribution of finishing times in our data set and changes in the ratio of women to men over time.

Reviewer #2, comment #2

In the methods section, I had a difficult time trying to understand how the demographics from the other 10 marathons would help you achieve your aim. The study adopted a pretty robust and complicated analysis, so every step of the data collection and analysis needs to be clearly explained. It is also important to report how many runners took part in each race you analysed and also split by age groups.

Our response #2.2

We have expanded our discussion on the data we collected to motivate how it is being used in more detail. We have also added a table that includes the number of runners in each race for each age group.

Reviewer #2, comment #3

I still think there are too many figures that do little to help you deliver your message.

Our response #2.3

We appreciate this feedback. We removed one of the figures from the supplemental info and we have expanded the discussion on several of our figures to make the message they are conveying more clear.

Reviewer #2, comment #4

In your results, you found that decreasing the qualifying times for the 35-39, 40-44, 45-49, 50-54 and 60-64 age groups would "optimise" the distribution of men and women across age groups. However, this approach, at the same time, substantially lowers the overall absolute number of qualifiers in the race, which will, in fact, exclude more runners. For this reason, I don't think your approach will "optimise" the qualifying times, as you would essentially exclude more people from taking part in the race.

Our response #2.4

We have updated the use of the word optimise to show we are trying to minimize our objective function which is to make the proportion of men and women similar. We also added a discussion of how this approach does exclude more runners and that other methods or objective functions could also be used that take the size of the field into consideration.

Reviewer #2, comment #5

The discussion should start by stating the aims of the study and providing a comprehensive summary of the main findings. The following paragraphs should then expand the main findings comparing your results with previous studies. I think the discussion is quite speculative and not supported by adequate evidence. For example, reference number 17, although interesting, is a blog post that probably was not peer-reviewed. There are also a couple of paragraphs (lines 199 to 214) including information not relevant to the study.

Our response #2.5

Thank you. We have removed some superfluous pieces from the discussion and have restructured to better fit the outline suggested here.

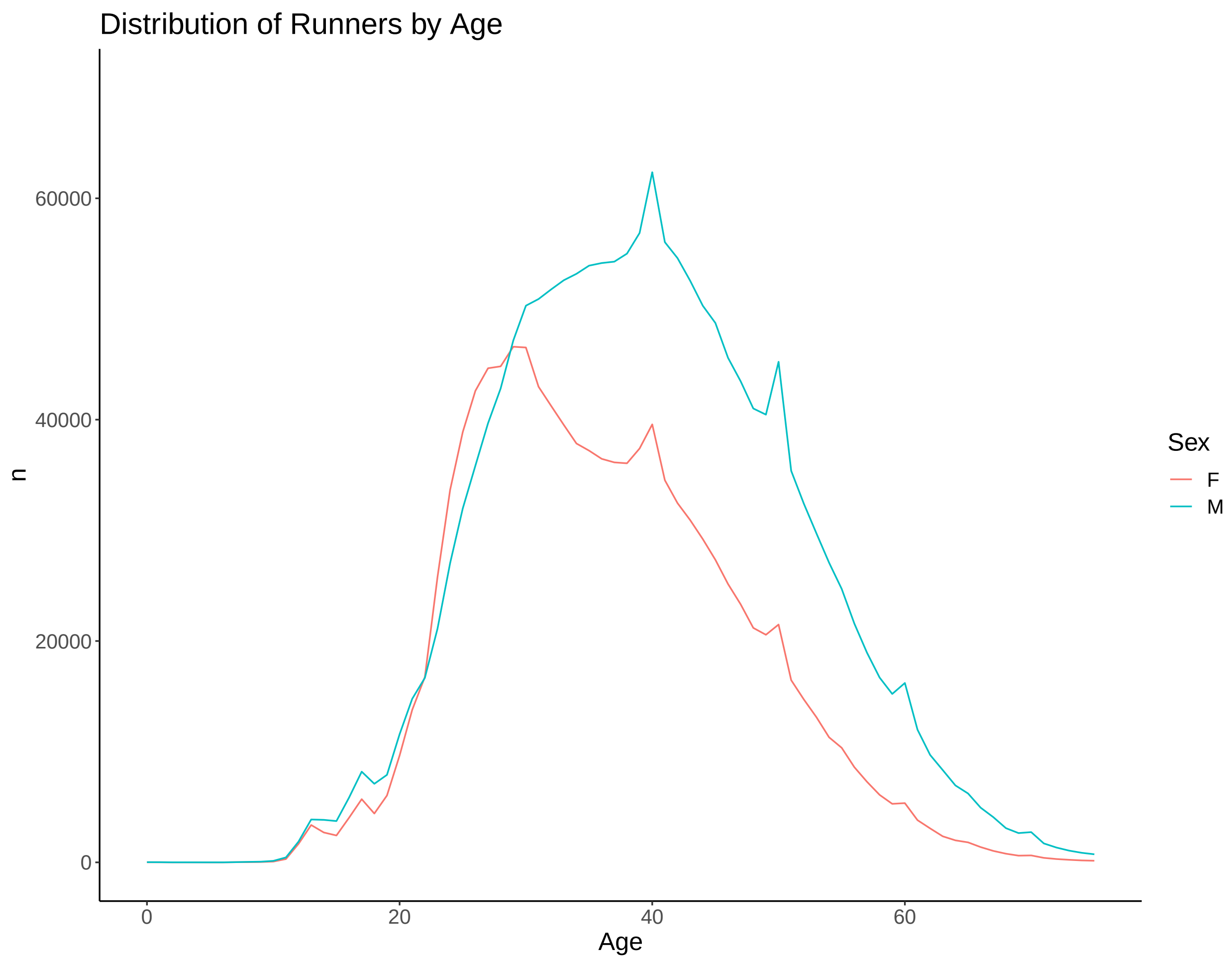

Figure 2: Distribution of men and women runners in our data set.

Reviewer #3, comment #1

Please give careful consideration to the statistical methods provided. Consider carefully reviewing several databases on running, like that of run repeat.

Our response #3.1

We appreciate this feedback and suggestion. We have updated our manuscript with a further discussion on the reason we have chosen this specific method for our optimization of the qualifying times. We have read through many of the studies at runrepeat.com and think their analysis is quite interesting. It is difficult to make direct comparisons between our analysis with their data as their data is not publicly available and much of their analysis centers around the average runner whereas we are focused on more competitive runners. We do observe similar trends in the distribution of runners in our data set and their database though. In the "State of Running 2019" post, they have a figure of the age distribution of female and male runners and observe that the distribution of female runners is more right skewed and male runners are more normally distributed. The distribution of runners by age in our data set shows very similar shapes to these plots (Fig 2).

They also show the percentage of women running by year which has a similar trend as Fig S3. The average finishing time by year for men vs women is shown in their post but it is not broken down into age groups. The only analysis they show comparing men and women in different age groups shows the pace of the average male and female runners at different distances. The average men's finishing time in our data varies by year between 4:18 - 4:30 and the average women's time is between 4:45 - 4:55 depending on the year. The runrepeat post does not provide raw results but, qualitatively, these ranges agree with their figure.

Analyzing finishing times for the average runner is not the aim of our study, however, comparing our data to their visualizations does provide evidence that our data is consistent with a much larger sample of race results.

Reviewer #3, comment #2

Addressing the Fairness question; reviewers struggled with this concept and I would decide on one clear path and stay with it. Reviewer 3 offers some ideas.

Our response #3.2

We have reworded the references to optimal/fair as suggested by Reviewer 3.

Reviewer #3, comment #3

Be clear on what your messaging is on the results. Editor note: I believe you've overplayed the grand outcomes and missed some key points, like the Boston qualifier really breaks down as a specific age. I believe reviewer 3 has offered some of the best advice to tackle the results issues, but it is a bit of work.

Our response #3.3

Thank you for this feedback. We have, accordingly, modified the discussion of our results to highlight the key points.
