# Supplementary material for: Data-driven evaluation of the Boston marathon qualifying times
Source: PLoS One. 2023 Apr 19;18(4):e0283851. doi: 10.1371/journal.pone.0283851 (PMC10115302; doi:10.1371/journal.pone.0283851)
Supplement: Attachment — Submitted filename: Review PONE-D-22-13227R1 GS.docx [file pone.0283851.s007.docx]

Review Greg Swinand

Data-driven evaluation of the Boston marathon qualifying times

**Recommendation: Revise and resubmit**

**Why this should be published**

Overall this is a very interesting paper and a very serious effort which raises all sorts of interesting questions for future research and contributes to the literature and so should be published.

The paper makes the point that current qualifying times of women relative to men by age group indicate women should get ‘relatively’ faster compared to men as they age.

The paper collects and cleans a large and likely robust (more on this later) dataset and this in and of itself is of interest to the scientific community as the data should be made available, and this alone might merit publication.

The paper uses a straightforward search algorithm to find the set of age-group women’s QTs that would minimize the total proportional differences between men’s and women’s age groups qualifying.

Overall, the authors do a good job or doing some sensitivity analysis and testing statistical differences based on their data.

The paper touches on many areas of research of interest, such as how age and gender affect endurance sport performance, fairness, sport participation by age and gender over time, etc.

**What needs to improve in order to be published**

While the overall effort is good, and the authors have addressed some concerns of the editor and previous comments of a reviewer, my opinion is they still have not gone far enough or addressed these adequately. I have also other concerns not raised previously.

**Statistical method**

A major issue is the sample and sampling method. The gold standard is random sampling, yet the authors say they chose by ‘geographic spread’ – is this good (probably not)? Is it random (definitely not)? Usually, in sampling, if trying to make inference about a population (such as the USA), then geographically representative proportions could be targeted, e.g., proportions of population by State. This would involve heavier weighting of more populous states. One might only want a uniformly geographic sample, if say, we were sampling 50 senators on how they would vote on something.

If random is not possible would some other matching or appropriate sampling method be better? These seem to be the ‘most common races’ to qualify for Boston, but how common? Can we know what % of qualifiers this would represent? What is the population of potential qualifiers? Are they actually mostly in States like California, Illinois, NY, etc? This is just not clear.

They then further carry out sensitivity checks by excluding one marathon and seeing the difference to the results. Line 128-34 gives that this might make 1 minute difference – is this significant? Moreover, however, this robustness check merely tests their sample to sensitivity WITHIN THEIR ALREADY (BIASed???) SELECTED SAMPLE. What we’d really like to know is how different this sample is to a population, not relative to itself minus one. They either need to have some way of finding covariates for marathons and marathoners included and excluded and seeing if these are matching or finding more data on dissimilar marathons, or give evidence the marathons excluded are so small they will not matter.

The remedy suggested here is several-fold.

Do we know something about runners and where they are from, nationally and in the races selected? Can we know what % of qualifiers come from these races? If this information is available, use it and discuss why this means the sample chosen is good. For example, we might be close to the population here—if more than 2/3s or ¾ of qualifiers are already coming from these races, then we’re a long way to actually having the population, rather than a sample.

If this isn’t available, some means of choosing some random races and checking statistically a) how differently they are from the current sample and b) how much the sample might change if they were included.

**Fairness**

There is a pandora’s box of issues with regards to ‘fairness’ and I think still more effort is needed here. The use of the word ‘optimal’ while in a mathematical sense is not linked to fairness still actually IS linked to some notion of fairness, as the authors have arbitrarily chosen an objective function to minimize – why not the sum of squared proportional differences? Why not weight by income for age group? Optimized in the pure mathematical sense means a chosen objective function. On the other hand, it can be interpreted more widely to mean ‘best’ – but who’s best? Semantically there is no difference between this looser understanding of ‘optimal’ and ‘fair’ or ‘best’. They should avoid the use of ‘optimized’ – and instead use something such as proportional-difference-minimized (or perhaps they can come up with some other way to name their outcome).

This is not intractable, nor should the paper get too much into this, but recognition of this and some discussion is needed and to be clear that this is ‘optimal’ only in the sense of having minimised an arbitrary objective function.

To show how this could go in many ways, they seem to indicate their proposed qualifying standard is ‘better’ if Boston used – why? What is the Boston Marathon’s objective function? What should it be?

Why not also minimize the proportional differences ‘across age groups’ for men and women combined, or separately? (In other words, what % of men and women 75-79 qualify relative to say 30-34?) My understanding is that the proposed approach minimises the proportional difference in qualifiers relative to men of the SAME age group—but what if both men AND women find it harder to qualify as they get older?

Further, they seem to indicate Boston might not have made their QTs based on evidence—do we know this? –maybe they had evidence that simplicity was better in some cases than (an undefined) fairness? We just don’t know.

**Results, outcomes and interpretations**

The results and proposed changes don’t really seem to change that much relative to what the authors claim as their original purpose is. Moreover, they seem to gloss this over, or else they’ve missed it.

Fig 2 has most all of the needed information of the paper and so it is useful to reproduce it here.

The ‘optimal’ new times would appear to trade-off 3,792 fewer women qualifying overall, 5,519 lose out on qualifying across the negative categories, but mostly in the 35-39 and 40-44 age cates, all to get 148 more women into 65-74 and 1579 into 18-34? It would appear the main redistribution is towards young women, no? Why is this ‘better’? Is it possible I have read this table wrong? In spite of the authors saying that the main thing ‘wrong’ with the current system being that it would appear women in older age categories are disadvantaged relative to men, their results overall on the face of it don’t really seem to bear this out; the main increase is in 18-34 year old women. Now granted in % terms the largest is 70-74 year olds.

(Moreover, I don’t understand the fact that all the % in the bottom row are negative but the (+/-) signs in the bottom ()’s seem consistent with row 2?.

I would have thought a more sophisticated constrained optimisation, holding the total number of women constant (for example), would have been possible.

This further also raises the question of whether the overall proportion of women qualifying is ‘harder’ or easier than for men; some mention and discussion of this should be done. (In other words, do 10% of top women qualify at the top races and 10% of men, roughly?, or is it 10,000 men and 10,000 women, and maybe that’s 5% and 10% of some pool of potential qualifiers?).

An interesting comparison here would be how the overall number of men and women changed in Boston when Boston changed the qualifying standards. How do these numbers compare to the proposed new changes? Are there other marathons that have changed similarly.

The authors then seem to gloss over this fact of the potential large increase in 18-34 women – for example:

1) they don’t mention this in the conclusion.
2) At line 187 they state, “Our results are consistent with those in [20] that found the 2013 Boston qualifying standards were too lenient for women 18-54 and too strict for women 55-80.” -- this seems in contradiction to their results – their results find QTs too strict for 18-34 AND 55-75 women.
3) The conclusions seem to indicate that the +1 minute for the 18-34 group found from the procedure should not be done; but the other proposed changes should be done.

At line 166 they seem to be over-interpreting their results:

“This is thought to be a result of the disproportionate obligations women still face in child rearing which could result in runners in these age groups being disproportionately comprised [in their training relative to?] of more serious and faster runners [17].”

Is this really true? There is just one (seemingly a very weak reference?) The statement would be much better written as “one interpretation suggested this to be a result of the disproportionate…..”

Moreover, it seems strange that this claimed child-bearing rationale is given for 35-39, and 40-44 women’s getting faster (and skewed distribution).

A quick google of this for fertility by mother’s age seems to indicate differently—the highest birth rates are for 20-30 years olds:

This seems to be a very charged and unsubstantiated claim which is at odds with basic fertility rate data – it may be true – but the authors need to either address this substantially or take this out.

Finally, the conclusions use language that is a bit unclear.

“By optimizing the proportion of qualifiers across all marathons and years”

Besides the already mentioned suggestion that ‘optimal’ is little better than ‘fair’ – what does ‘optimising the proportion’ mean? What actually was done was minimize proportional differences between gender for each age cohort, no? Some more precise language and description is required here.

“Our method allows the qualifying times to be supported by data instead of being set on a sliding scale”; again, do we know the sliding scale wasn’t set using data? Say something more precise about what was done – e.g., using the large data set covering x% of all qualifiers from past 10 years (or whatever that should say).

“leading to a proportionally more representative group of qualifiers across gender and age.”

Again, it isn’t clear what this means and is seemingly imprecise—representative on what?---we need to have some prior of the ‘true’ population proportion. Usually, when we say “representative” we mean that a sample is constructed to be proportionally matched on some criteria, such as proportion of males and females, age cohort, in a population—the population proportion is known (e.g., Census data). But this isn’t what is going on here – either make the case of why this is ‘representative’ of a population proportion or use some other language.
